# Video Suppression Head Impulses and Head Impulses Paradigms in Patients with Vestibular Neuritis: A Comparative Study

**DOI:** 10.3390/healthcare10101926

**Published:** 2022-09-30

**Authors:** Leonardo Manzari, Amaranta Soledad Orejel Bustos, Alessandro Antonio Princi, Marco Tramontano

**Affiliations:** 1 MSA ENT Academy Center, 03043 Cassino, Italy; 2 Fondazione Santa Lucia IRCCS, 00179 Rome, Italy; 3 Department of Movement, Human and Health Sciences, University of Rome “Foro Italico”, 00135 Rome, Italy

**Keywords:** vestibulopathy, vestibular system, SHIMP, HIMP, vHIT

## Abstract

Background: This study aims to explore the clinical relevance of the Suppression Head Impulse Paradigm (SHIMP) to better understand if it represents an additional clinical value compared to the Head Impulse Paradigm (HIMP) in patients with vestibular neuritis (VN) in different stages of the disease. Methods: From January 2020 to June 2022, patients with unilateral VN were found in a database of an ENT vestibular clinic. Clinical presentation, vestibular test outcomes, therapy, and recovery were examined in medical records. Results: A total of 42 patients (16 Females, mean age 51.06 ± 12.96; 26 Male, mean age 62.50 ± 9.82) met the inclusion criteria and were enrolled in the study. The means of the VOR gain for both paradigms were respectively 0.38 ± 0.12 (SHIMP) and 0.46 ± 0.13 (HIMP) at T0 and 0.55 ± 0.20 (SHIMP) and 0.64 ± 0.19 (HIMP) at T1 for the lesional side. For the HIMP, the gain value <0.76 identified the affected side of VN with 100% sensitivity (92–100) and 100% specificity (91–100). For the SHIMP, the gain value <0.66 identified the affected side of VN with 100% sensitivity (92–100) and 100% specificity (91–100) and an AUC of 1.0 (0.96–1.0, *p* < 0.0001). Conclusion: The SHIMP paradigm has a diagnostic accuracy equal to the classic HIMP paradigm in patients with VN. The assessment of VOR slow phase velocity and vestibulo-saccadic interaction in patients with VN could be easier with the use of the SHIMPs paradigm. SHIMPs paradigm provides helpful information about the evaluation of VOR slow phase velocity and vestibulo-saccadic interaction as new recovery strategies in patients with VN.

## 1. Introduction

To assess the canal vestibular function in patients with acute vestibular syndrome (AVS) it is important to differentiate Vestibular Neuritis (VN) from a cerebellar or brainstem stroke [1]. Indeed, combining the video Head Impulse Test (vHIT) with the HINTS protocol (Head-Impulse—Nystagmus—Test-of-Skew) could help clinicians in finding false positives for stroke [2]. The evaluation of the Vestibulo Ocular Reflex (VOR) through the vHIT is nowadays a routine clinical test to measure the VOR gain and the correct eye movements during an unpredictable passive head turn [3]. Specifically, two evaluation paradigms are available to study the canal vestibular function, the Head Impulse Paradigm (HIMP) and the Suppression Head Impulse Paradigm (SHIMP). In the first case, with 100% of sensitivity and specificity, the clinician can assess the functional condition of each semicircular canal, while the person maintains the fixation on an earth-fixed target during small, abrupt, passive, unpredictable impulsive turns following the plane of the tested canal [4]. In the second paradigm, with the same reliability, it is possible to assess the function of the horizontal semicircular canal VOR slow phase velocity when the person maintains the fixation on a head-fixed target during small, abrupt, passive, unpredictable impulsive turns in the horizontal plane [4]. In the case of vestibulopathy, during the HIMP a covert or overt saccade appears during the test, while during the SHIMP the patient does not make a corrective saccade (a “SHIMPs’’saccade) in both unilateral and bilateral vestibulopathy [5,6,7]. Recently, a study [8] reported the presence of anticompensatory saccades (AcS) also on the healthy side in patients with VN during the HIMP in the acute phase. Indeed, the presence of AcS could be a sign of VOR altered function, similar to what happens on the affected side. Conversely, the presence of the AcS during the SHIMP is a physiological response to regain the target at the end of the head rotation and their reappearance after an AVS is the clinical sign of a probable recovery of the VOR [9]

Recently it has been argued that SHIMP gives more precise information on the VOR slow phase velocity value compared to HIMP because the evaluation of the gain is not affected by covert saccades [9,10]. Several studies [11,12] have already suggested the use of vHIT with HIMP to diagnose a VN in the acute phase and as a valid tool to diagnose peripheral vestibulopathy in the different stages of the disease [3]. A recent systematic review [13] highlighted the usefulness of the SHIMP in diagnosing a VOR alteration in patients with vestibulopathy suggesting to evaluate with new studies if this new paradigm could replace the HIMP in both the acute and chronic phases of vestibulopathy. For these reasons, we hypothesize that SHIMP could be compared, in terms of usefulness, to the HIMP in evaluating eye movement and visuo-vestibular interaction strategy alteration in patients with acute and chronic VN. Indeed, anti-compensatory saccades and their relationship with the vestibular input could be a useful mechanism for minimizing subjective symptoms after acute vestibular loss through efficient interaction between the visual and vestibular systems. Chen F. and colleagues [14] performed an association analysis of HIMP and SHIMP quantitative parameters and underlined that the two vHIT-based paradigms potentially provide a comprehensive assessment of vestibular loss and function by measuring VOR gain in patients with VN evaluated within 7 days from the onset.

To date, no studies have compared the simultaneous application of SHIMPs and HIMPs paradigms in different stages of the disease of VN. Thus, this study aims to investigate the clinical usefulness of the SHIMP in comparison with the HIMP in the acute phase and during the follow-up in a large sample of patients with superior VN.

## 2. Materials and Methods

### 2.1. Study Design

This retrospective study compares SHIMP and HIMP to assess the VOR gain values of the horizontal canal across the various stages of the VN. The STROBE (Strengthening the Reporting of Observational Studies in Epidemiology) principles were followed when conducting the study. All patients gave written consent to publish the results obtained from their clinical examinations and instrumental tests.

### 2.2. Setting

Medical records of patients with a diagnosis of VN in the early stages of the disease (from the first hours to six weeks since the AVS) and still symptomatic who were admitted to the ENT MSA Academy Center from January 2020 to June 2022 were reviewed.

### 2.3. Participants

Patients who had a diagnosis of VN in the early stages (within 72 h of the AVS) met the inclusion criteria. Patients with another vestibular diagnosis (more than six weeks since the AVS, Ménière’s Disease, bilateral vestibular loss, vestibular migraine, benign paroxysmal positional vertigo, acoustic neuroma, superior semicircular canal dehiscence syndrome), somatic or psychiatric disorders or neurological diseases were not included in the study. All patients underwent a vestibular evaluation that included a bedside Head Impulse Test + video-Head Impulse Test, Air Conducted Sound, and Bone Conducted Vibration Cervical and Ocular VEMPs, an evaluation of the horizontal and vertical semicircular canals, and an audiological assessment.

The following criteria were used to diagnose VN: (a) a history of acute onset of severe, protracted, rotatory vertigo, nausea, and postural imbalance; (b) on clinical examination, the presence of horizontal spontaneous nystagmus with a rotational component toward the unaffected ear (fast phase); (c) abnormal bed-side HIT showing an ipsilateral deficit of the horizontal semicircular canal; [4]; (d) alterations in the cervical and ocular VEMPs results compatible with the diagnosis of Vestibulopathy (e) an MRI was performed at least 48 h after the AVS onset, and data saved in the medical records of the patients [15,16]. After 30 days and during the acute phase (approximately 72 h), patients were examined.

### 2.4. Video Head Impulse Test

Using horizontal vHIT (OtosuiteV^®^, GN Otometrics, Taastrup, Denmark), the semicircular canals’ function was assessed. Gain occurs during the VOR slow phase in the HIMP and SHIMP paradigms. The gain value of 0.76 has 100% specificity (48–100) and 100% sensitivity in identifying the affected side of Unilateral VN (74–100). The area under the curve (AUC) during HIMP is 1.0 (0.81–1.0, *p* < 0.0001), and VOR gain is the ratio of that value [4].

With 100% sensitivity (48–100) and 100% specificity (74–100) and an AUC of 1.0 (0.81–1.0, *p* < 0.0001), SHIMP gains (0.66) detected the affected side of Unilateral VN [9]. Comparatively, Anticompensatory Saccades (AcS) or fast eye movement on the contralesional side were defined as saccades (peak velocity exceeding 50°/s) in the direction of the head movement. Compensatory saccades on the affected side were defined as saccades in the direction of eye movement. AcS start was at 10°/s, latency was the difference between head impulse and AcS start. AcS occurrence rate was the percentage of impulses with AcS [17].

### 2.5. Statistical Analysis

Statistical analyses were carried out using the IBM SPSS Statistic Software Version 23, IBM Corp., Armonk, NY, USA. Based on the skewness and kurtosis statistics, the variables were judged to be normally distributed when the skew and kurtosis levels were <|2.0| and <|9.0|, respectively [18,19]. Descriptive statistics were calculated for all variables of interest, and data are presented as mean and standard deviation. A paired samples *t*-test was performed to analyze differences in the VOR gain during SHIMP and HIMP paradigms in the acute phase (T0) and during a follow-up (T1). Diagnostic accuracy for the two vHIT paradigms was performed using the Receiver Operating Characteristic (ROC) curve analysis with MedCalc software (Ostend, Belgium). The significance level was set at *p* < 0.05.

## 3. Results

Forty-two patients (16 females, mean age 51.06 ± 12.96; 26 male, mean age 62.50 ±9.82) met the inclusion criteria and were enrolled in the study. Clinical and demographic characteristics are reported in Table 1.

Based on skewness and kurtosis statistics, data were normally distributed for SHIMP in the lesional (−1.27 and 1.95) and contralesional side (−0.27 and −0.53), respectively; and for HIMP in the lesional (−1.03 and 2.33) and contralesional side (−0.85 and 2.43).

The means of the VOR gain for both paradigms were respectively 0.38 ± 0.12 (SHIMP) and 0.46 ± 0.13 (HIMP) at T0, and 0.55 ± 0.20 (SHIMP) and 0.64 ± 0.19 (HIMP) at T1 for the lesional side. For the contralesional side, 0.85 ± 0.12 (SHIMP) and 0.91 ± 0.14 (HIMP) at T0, and 0.91 ± 0.12 (SHIMP) and 0.99 ± 0.13 (HIMP) at T1.

**Figure 1:**
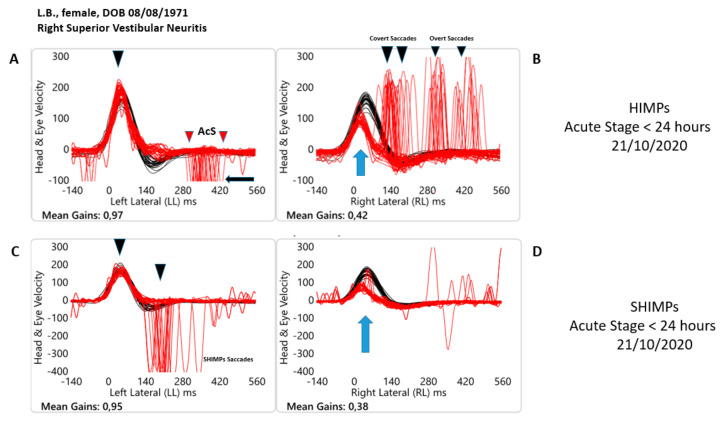
Objective measures of horizontal semicircular canal function at the time of the attack (<24 h) in both paradigm HIMPs (**A**,**B**) and SHIMPs (**C**,**D**) with video Head Impulse test Technology for one patient with acute unilateral (right) superior vestibular neuritis. Each panel shows a superimposed time series of head velocity (black for the head impulses) and the corresponding eye velocity (red) for the lateral canal dynamic function. The signs of head velocity for leftward impulses and of eye velocity for rightward impulses have been inverted to allow for easier comparison. Normal horizontal Vestibulo Ocular Reflex (VOR) gains are approximately 0.7–1.0 [4]. At the time of the attack (**A**,**B**) HIMP paradigm. For rotations to the affected side, eye velocity is substantially less than the corresponding head velocity during the head turn so the VOR is significantly less, VOR gain is 0.42, then for head turns to the healthy side, VOR gain is 0.97. There is a shower of compensatory (Covert + Overt) saccades during and at the end of the head turn (black arrow). When the head is turned to the left contralesional side, anti-compensatory saccades (AcS) eye movements in the direction of the head movement can be observed (red arrow). At the time of the attack (**C**,**D**) SHIMP paradigm. In this paradigm for rotations toward the affected side, eye velocity is substantially less than the corresponding head velocity (red traces vs. black traces). Note how during the head turn, the slow phase eye velocity of the VOR is significantly less also in comparison with the same value in the HIMP paradigm [VOR gain is 0.38]. For head turns to the healthy side, VOR gain is 0.95. The blue arrows indicate the reduced angular velocity of the slow phase of the VOR. When the head is turned to the left contralesional, healthy side, anti-compensatory overt SHIMPs saccades at the end of the head turn (black arrow, **C**) eye movements in the direction of the head movement can be observed. In sharp contrast, at the time of the attack, when the head is turned toward the right affected side, note how no anti-compensatory SHIMPs saccades can be found.

Participants showed a significant larger VOR gain values with HIMP when compared with SHIMP (∆ 0.08 ± 0.08) at T0 for the lesional, *t*(41) = −6.54, *p* < 0.001, *d* = 0.60; and contralesional sides, *t*(41) = −3.34, *p* = 0.002, *d* = 0.47 (see Figure 1).

**Figure 2:**
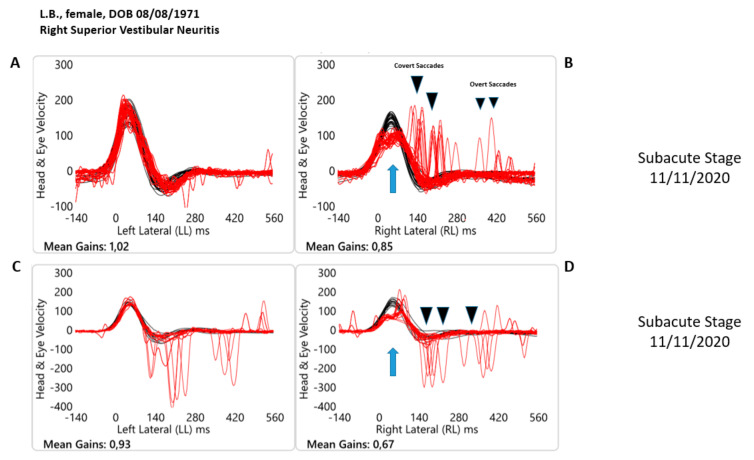
Objective measures of horizontal semicircular canal function at the time at the subacute stage (10 days later the onset of the acute vestibular syndrome) in both paradigm HIMPs (**A**,**B**) and SHIMPs (**C**,**D**) with video Head Impulse test Technology for one patient with acute unilateral (right) superior vestibular neuritis. Each panel shows a superimposed time series of head velocity (black for the head impulses) and the corresponding eye velocity (red) for the lateral canal dynamic function. The signs of head velocity for leftward impulses and of eye velocity for rightward impulses have been inverted to allow for easier comparison. Normal horizontal Vestibulo Ocular Reflex (VOR) gains are approximately 0.7–1.0 [4]. Subacute stage (**A**,**B**) HIMP paradigm. For rotations to the affected side, slow phase eye velocity is back within the limits of the norm but substantially less than the corresponding head velocity during the head turn so the VOR is slightly less, VOR gain is 0.85, then for head turns to the healthy side, VOR gain is 1.02. There is still a shower of compensatory (Covert + Overt) saccades during and at the end of the head turn (black arrow). When the head is turned to the left contralesional side, anti-compensatory saccades (AcS) eye movements in the direction of the head movement have disappeared (see Figure 1, panel A, for comparison). Subacute stage (**C**,**D**) SHIMP paradigm. In this paradigm for rotations toward the affected side, eye velocity is still substantially less than the corresponding head velocity (red traces vs. black traces). Note how during the head turn, the slow phase eye velocity of the VOR is significantly less also in comparison with the same value in the HIMP paradigm (VOR gain is 0.67). For head turns to the healthy side, VOR gain is the same, 0.93. The blue arrows indicate the reduced angular velocity of the slow phase of the VOR. When the head is turned to the left contralesional, healthy side, anti-compensatory overt SHIMPs saccades at the end of the head turn (black arrow, **C**) eye movements in the direction of the head movement can be observed. In sharp contrast with the acute stage, on this occasion, when the head is turned toward the right affected side, note how anti-compensatory SHIMPs saccades have reappeared.

**Figure 3:**
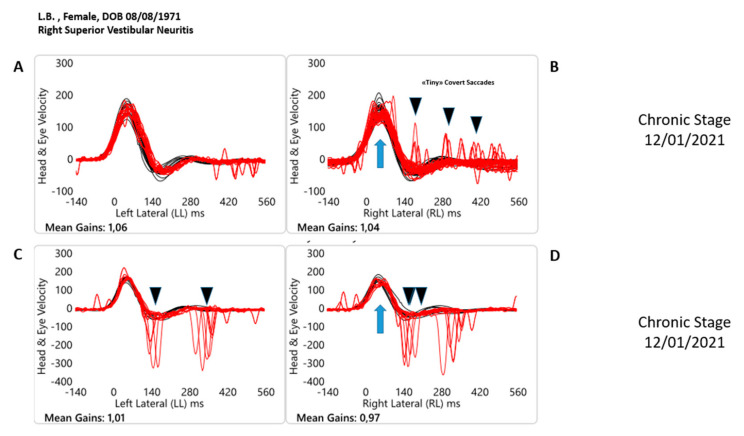
Objective measures of horizontal semicircular canal function at the time at the chronic stage (50 days later than the onset of the acute vestibular syndrome) in both paradigm HIMPs (**A**,**B**) and SHIMPs (**C**,**D**) with video Head Impulse test Technology for one patient with acute unilateral (right) superior vestibular neuritis. Each panel shows a superimposed time series of head velocity (black for the head impulses) and the corresponding eye velocity (red) for the lateral canal dynamic function. The signs of head velocity for leftward impulses and of eye velocity for rightward impulses have been inverted to allow for easier comparison. Normal horizontal Vestibulo Ocular Reflex (VOR) gains are approximately 0.7–1.0 [4]. Chronic stage (**A**,**B**) HIMP paradigm. For rotations to the affected side, slow phase eye velocity is back within the limits of the norm, VOR gain is 1.04, then for head turns to the healthy side, VOR gain is 1.06. There is still a shower of tiny compensatory (Covert + Overt) saccades during and at the end of the head turn (black arrow). When the head is turned to the left contralesional side, anti-compensatory saccades (AcS) eye movements in the direction of the head movement have disappeared (see Figure 1, panel A, for comparison). Chronic stage (**C**,**D**) SHIMP paradigm. In this paradigm for rotations toward the affected side, slow phase of the eye velocity is back within the limits of the norm (red traces vs black traces). Note how during the head turn, the eye slow phase velocity of the VOR is within the limits of the norm in comparison with the same value in the HIMP paradigm [VOR gain is 0.97]. For head turns to the healthy side, VOR gain is exactly the same, 1.01. The blue arrows indicate the return to normal angular velocity of the slow phase of the VOR when the head, in both paradigms, is turned toward the affected side. When the head is turned to the left contralesional, healthy side, anti-compensatory overt SHIMPs saccades at the end of the head turn (black arrow, **C**) eye movements in the direction of the head movement can be observed. In sharp contrast with the acute stage, on this occasion, when the head is turned toward the right affected side, note how anti-compensatory SHIMPs saccades have reappeared.

Similarly, significant larger VOR gain values for HIMP were found at T1 for the lesional, *t*(41) = −7.87, *p* < 0.001, *d* = 0.45; and contralesional sides *t*(41) = −5.52, *p* < 0.001, *d* = 0.63 when compared to SHIMP (∆ 0.09 ± 0.01) (see Figure 2 and Figure 3). VOR gain descriptive statistics are reported in Table 2.

For the HIMP, the gain value <0.76 identified the affected side of VN with 100% sensitivity (92–100) and 100% specificity (91–100). For the SHIMP, the gain value <0.66 identified the affected side of VN with 100% sensitivity (92–100) and 100% specificity (91–100) and an AUC of 1.0 (0.96–1.0, *p* < 0.0001). During both paradigms, the VOR gain represented the ratio of the area under the curve (AUC) of 1.0 (0.95–1.0, *p* < 0.0001).

## 4. Discussion

This study aimed to investigate the clinical usefulness of the SHIMP paradigm in comparison with HIMP in the acute phase and during the follow-up in patients with VN.

HIMP and SHIMP are two different paradigms for the clinical evaluation of the state of health of the vestibulo-oculomotor reflex (HIMP) and in addition to this also for the evaluation of the visual and vestibular interaction (SHIMP).

Our results confirm that both paradigms had a 100% sensitivity and specificity in the evaluation of the VOR function in patients with VN during the acute phase and after at least 30 days from the onset. Indeed, the mean of the VOR gain values was <0.76 for the HIMP and <0.66 for the SHIMP paradigms for the affected side. In the same way during acute and subacute or chronic stages, the paradigms present among themselves have different values of the VOR gain.

The angular velocity of the slow phase of the VOR in the HIMP paradigm presents values greater than those highlighted with the SHIMP and this seems to confirm the hypothesis that the contribution of the covert saccades is decisive in recording that type of value in the case of the classic paradigm proposed by Halmagyi and Curthoys [4,5]. For this reason, the execution of the SHIMP paradigm appears to be able to better evaluate the effective value of the VOR gain. In both acute and chronic stages, the paradigms present different values of the VOR gain. The difference between the two paradigms of the VOR gain mean at T0 is 0.08 ± 0.01 and 0.09 ± 0.01 at T1. From a clinical point of view, this result is a relevant and interesting fact because the evaluation of the angular velocity of the slow phase in both paradigms is lower than the physiological range and at the same time both paradigms can highlight this deficit during a VN.

Interestingly, this difference was also found for the contralesional side at T0 (0.06 ± 0.02) and T1 (0.08 ± 0.00). Indeed, since its first description, the SHIMP paradigm has highlighted some peculiarities due to the difference in execution compared to the HIMP paradigm. The two peculiarities were essentially characterized in healthy people by the presence of anticompensatory saccades at the end of the head turns and these saccades were considered indicators of vestibular function.

MacDougall and colleagues [4] have demonstrated that in SHIMPs, the gain of the VOR was slightly lower than HIMPs gains also on the contralesional side as confirmed by our results. From our point of view, we can hypothesize that this difference can be due to two kinds of factors.

Firstly, healthy people can, after a delay, suppress their slow phase eye velocity response elicited by semicircular canal stimulation in the SHIMP paradigm. This phenomenon has been well described by Crane and Demer [20]. For this reason, it is necessary that the person executes a refixation saccade in the direction of the movement of the head at the end of the head turns to bring the aim back to the fovea.

Secondly, the de-saccading algorithm [21], which vHIT technology uses to remove the catch-up saccades during the time window for VOR gain measurements, may be responsible for this systematic difference between the two paradigms. Our results confirm MacDougall and Colleagues [21] hypothesis that “...this, in turn, would be an additional argument in favor of the new SHIMP paradigm, as it usually delays any saccades until after the end of the head impulse….” These two characteristics that differentiate the new paradigm from the older are particularly intriguing for researchers and clinicians. Therefore, it appears reductive to consider only the compensatory saccades data in the clinical evaluation process of a patient affected by AVS.

### Strengths and Limitations

To the best of our knowledge, this is the first study with a large sample aiming to compare the two paradigms in patients with VN, but further studies are needed and routine application will be necessary to acquire more experience about the clinical utility of SHIMP at the bedside. We are aware that this study presents some limitations that should be mentioned. First, this is a retrospective study, with intrinsic potential bias. Second, we did not recruit children. Third, vHIT was performed using only the OtosuiteV^®^ tool but more instruments should be compared.

## 5. Conclusions

Our results confirm that the SHIMP paradigm has a diagnostic accuracy equal to the classic HIMP paradigm in patients with VN. This confirmation comes from the data of the angular velocity of the slow phase of the VOR and therefore the SHIMP paradigm can replace HIMP with the same clinical efficacy. The clinician can formulate a diagnosis and follow the trend of the recovery of the VOR gain even just using the SHIMP paradigm.

## Figures and Tables

**Table 1:** Clinical characteristics of the VN population.

Demographics	
No. of patients	42
Age, years, mean ± SD	58.14 ± 12.32
Sex, male	26 (61.9)
**Affected side**	
Left	19 (45.2)
Right	23 (54.8)
**Time since neuritis**	
≤24 h	15 (35.71)
Between 24 and 48 h	12 (28.57)
Between 48 and 72 h	15 (35.71)
**Follow-up**	
30 days	9 (21.4)
Between 30 and 60 days	24 (57.1)
Greater than 60 days	9 (21.4)

*Note:* Data are n (%) unless otherwise indicated. Abbreviations: VN, vestibular neuritis.

**Table 2:** Descriptive statistics of Vestibulo Ocular Reflex gain values across time.

Paradigm	T0		T1	
	Lesional Side	Contra Lesional Side	Lesional Side	Contra Lesional Side
SHIMP	0.38 ± 0.12	0.85 ± 0.12	0.55 ± 0.20	0.91 ± 0.12
HIMP	0.46 ± 0.13	0.91 ± 0.14	0.64 ± 0.19	0.99 ± 0.13
**Δ** HIMPvsSHIMP	0.08 ± 0.08	0.06 ± 0.12	0.09 ± 0.07	0.08 ± 0.09

## Data Availability

The data presented in this study are available on request from the corresponding author.
